# Computational drug repositioning with attention walking

**DOI:** 10.1038/s41598-024-60756-6

**Published:** 2024-05-02

**Authors:** Jong-Hoon Park, Young-Rae Cho

**Affiliations:** 1 Division of Software, Yonsei University Mirae Campus, Wonju-si, 26493 Gangwon-do Korea; 2 Division of Digital Healthcare, Yonsei University Mirae Campus, Wonju-si, 26493 Gangwon-do Korea

**Keywords:** Machine learning, Network topology

## Abstract

Drug repositioning aims to identify new therapeutic indications for approved medications. Recently, the importance of computational drug repositioning has been highlighted because it can reduce the costs, development time, and risks compared to traditional drug discovery. Most approaches in this area use networks for systematic analysis. Inferring drug-disease associations is then defined as a link prediction problem in a heterogeneous network composed of drugs and diseases. In this article, we present a novel method of computational drug repositioning, named drug repositioning with attention walking (DRAW). DRAW proceeds as follows: first, a subgraph enclosing the target link for prediction is extracted. Second, a graph convolutional network captures the structural features of the labeled nodes in the subgraph. Third, the transition probabilities are computed using attention mechanisms and converted into random walk profiles. Finally, a multi-layer perceptron takes random walk profiles and predicts whether a target link exists. As an experiment, we constructed two heterogeneous networks with drug-drug similarities based on chemical structures and anatomical therapeutic chemical classification (ATC) codes. Using 10-fold cross-validation, DRAW achieved an area under the receiver operating characteristic (ROC) curve of 0.903 and outperformed state-of-the-art methods. Moreover, we demonstrated the results of case studies for selected drugs and diseases to further confirm the capability of DRAW to predict drug-disease associations.

## Introduction

Although there has been enormous outgrowth over the decades in pharmacology, biology, and genomics, developing new drugs can be a lengthy, expensive, and risky process^1,2^. It takes approximately 15 years and costs more than 1.5 billion dollars^3–5^. Investments in drug discovery have increased recently; however, the number of new drugs approved by the US Food and Drug Administration (FDA) is declining. Therefore, drug repositioning, also referred to as drug repurposing, has recently gained attention. It is one of the approaches to drug discovery that identifies new therapeutic indications for medications already confirmed by the FDA^6^. Recently, several cases of successful drug repositioning have been reported. For instance, thalidomide was developed as a sedative that was especially effective for morning sickness but had the problem of causing birth defects in pregnant women, so thalidomide was prohibited from sale. However, it was later discovered to be effective in the treatment of multiple myeloma and leprosy^7^.

In the sense of the growing drug repositioning field, computational drug repositioning is attracting interest from biomedical researchers and pharmaceutical companies^8–10^. It efficiently guides the priority of pairs of drugs and diseases so that drug discovery can be accelerated compared to traditional procedures. Computational approaches can integrate various types of genomic information, such as protein structure, sequence, and phenotype, to improve accuracy. Recently, most computational drug repositioning techniques have used networks composed of drugs, diseases, or related elements^11,12^. These networks can be organized based on biomedical features such as the chemical structures of drugs. The major advantage of using networks is that we can effectively manage and analyze data at the system level.

The drug repositioning problem can be viewed as a link prediction problem as we construct a network of drugs and diseases. Link prediction in networks (or graphs) is significant in diverse areas. It has already been adopted in recommendation systems^13^, citation networks^14^, and protein–protein interaction networks^15^. The link prediction problem can be solved successfully using simple heuristics, such as the Adamic-Adar (AA)^16^, Katz index^17^, and PageRank^18^. However, these heuristics have clear practical limitations in that they cannot be applied to all universal networks.

With the advancement of deep learning models and improved computing performance, methods adopting graph neural networks (GNNs)^19^ have been proposed in recent years. They perform well in graph representation learning, node classification, graph classification, and link prediction^20^. GNNs run not only for data in Euclidean space, but also for graph structures represented in non-Euclidean space. A GNN encodes hidden structural features that can be extracted from the topology of the input network^21^. It learns node representations based on information propagated from a node to its neighbors by message-passing rules, such that nodes sharing similar neighborhoods become similar entities.

In this study, we propose a novel method for computational drug repositioning, called Drug Repositioning with Attention Walking (DRAW), inspired by Walk Pooling^22^. To predict drug-disease associations, we constructed a heterogeneous network with drug-drug similarities, disease-disease similarities, and known drug-disease associations. A heterogeneous network is defined as a graph consisting of two or more types of nodes and their links, whereas a homogeneous network is composed of a single type of nodes and their links. Next, a subgraph enclosing the target link for prediction is extracted. This process transforms the link prediction problem into a graph-classification problem. To learn the structural features of the nodes in the subgraph, a graph convolutional network (GCN)^21^, which applies the concept of convolutional neural networks (CNNs)^23^ to a GNN, is used. Given these representations from the GCN, the attention mechanism^24^ is applied to reconstruct a transition probability matrix and compute the random walk profile. Finally, the features computed from the random walk profile are fed into a multilayer perceptron (MLP)^25^ to generate a score of the subgraph, which indicates a prediction score for the target link. Our experimental results demonstrated that DRAW outperformed its competitors, achieving an area under the ROC curve (AUC) score higher than 0.9.

The main contributions of this work are summarized as follows:

- We proposed a graph-based deep learning method using a GCN that can predict drug-disease associations accurately.
- We verified that a novel random walk method using attention mechanisms outperformed state-of-the-art methods for link prediction.
- We demonstrated that drug-drug similarities measured using ATC codes were more effective at drug repositioning than similarities based on chemical structures.

## Methods

**Figure 1:**
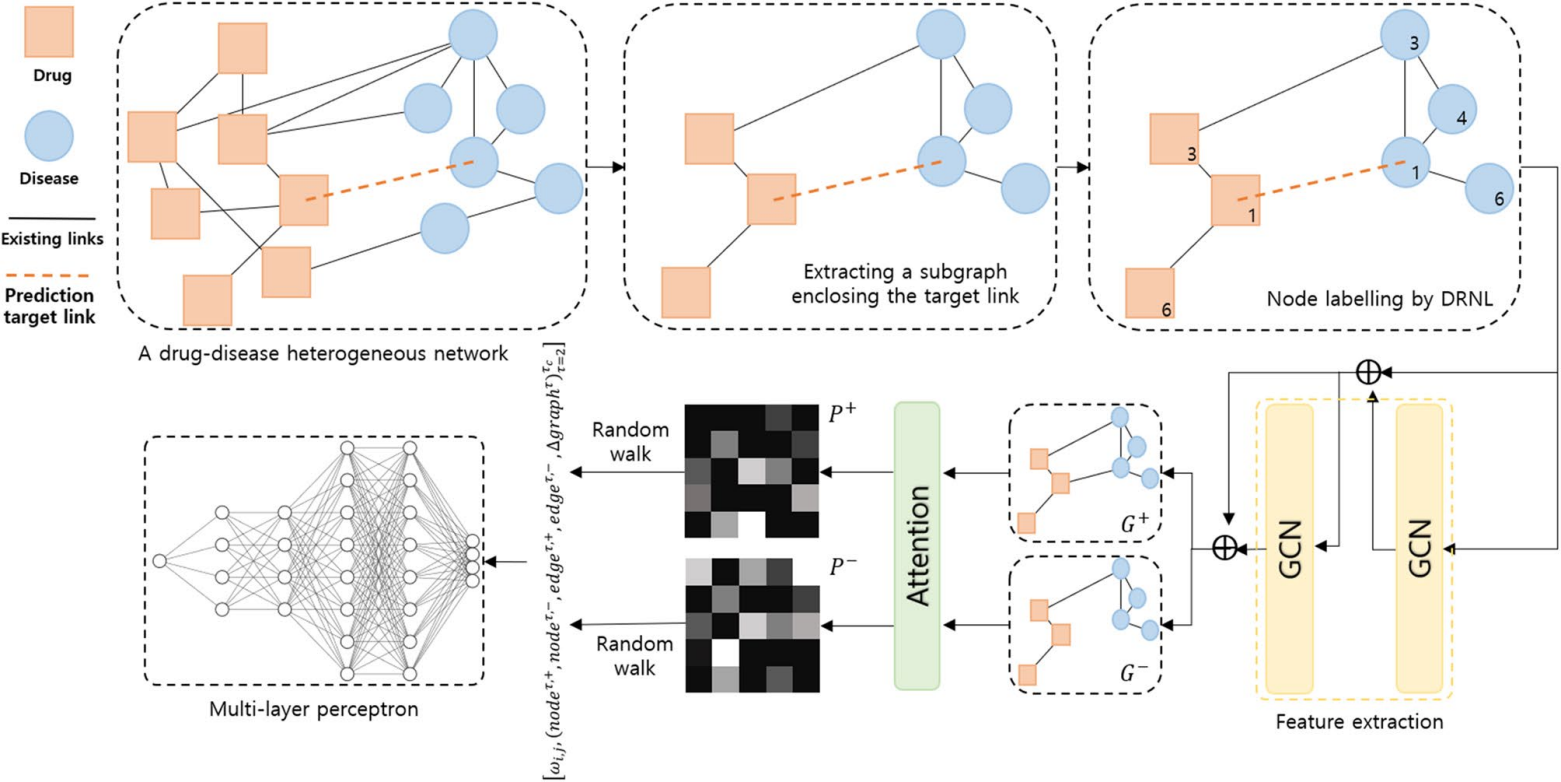
The workflow of the proposed model, DRAW. A subgraph composed of the nodes of the target link and their neighbors is extracted from the original drug-disease heterogeneous network. Nodes in the subgraph are labeled by DRNL and fed into the GCN. Transition probabilities ${{\varvec{P}}}^{+}$ and ${{\varvec{P}}}^{-}$ are computed by the attention conducted on ${{\varvec{G}}}^{+}$ and ${{\varvec{G}}}^{-}$ with the features from the GCN. After a random walk is completed, all features are used as input of MLP, a binary graph classifier.

In this section, we introduce our computational drug repositioning method, DRAW, which predicts drug-disease associations through the following steps. First, a drug-disease heterogeneous network is constructed in the form of an undirected, unweighted graph. Second, the subgraph enclosing the target link for prediction is extracted. This is used as the input for the binary graph classifier. Third, nodes in the subgraph are labeled as the Dual Radius Node Labeling (DRNL) scheme. Fourth, a GCN is adopted to extract the structural features of the subgraph. Fifth, a random walk profile is computed using attention mechanisms. Sixth, the features of the node-, edge-, and graph-level are calculated using the random walk profile. Finally, the graph classifier takes all features as inputs to score the subgraph for the purpose of predicting the presence of the target link. Overall, we enhanced the methodology to be applied to drug repositioning, incorporating refined network construction and parameter tuning into the original process of WalkPooling^22^. Figure 1 shows the workflow of DRAW.

### Network construction

A drug-disease heterogeneous network is structured by connecting drug pairs, disease pairs, and known drug-disease associations. However, including all drug-drug similarities and disease-disease similarities requires considerable memory usage and time. Thus, we selected drug pairs (and disease pairs) in order of the highest similarities as edges and adjusted the network density. We set the hyperparameters ${\mathcal{D}}_{dr}$ and ${\mathcal{D}}_{di}$ to represent density thresholds of the drug and disease networks, respectively. For the drug network, node pairs are selected up to $0.01 \times D_{dr} \times N_{dr} \times \left( {N_{dr} - 1} \right) \div 2$ where ${\mathcal{N}}_{dr}$ is the number of drugs. The disease network is the same as the drug network. In our experiment, we set densities ${\mathcal{D}}_{dr}$ and ${\mathcal{D}}_{di}$ to 4, empirically. The effect of network densities will be further discussed in the Results section. All edges in this heterogeneous network were weighted as 1 to create an unweighted graph.

### Subgraph extraction

To extract the subgraph enclosing the target link for prediction, a k-hop subgraph was sampled. According to previous reports^22,26^, the existence of a link between two nodes depends on the connectivity of their close neighbors. For instance, link prediction using the Jaccard index showed good performance, even though it considered only the closest neighbors. In agreement with these studies, we adopted $k=2$ in our experiment. When a subgraph is extracted, the link prediction problem can be redefined as a graph classification problem. If a graph is classified as true, then the target link is predicted positively and vice versa.

### Node labelling by DRNL

To create topological features of the subgraph, we used the DRNL algorithm proposed by Zhang and Chen^26^. The result involves the extent to which each node is separated from the target nodes connected by the target link. This is formulated as follows:

$$DRNL(i) = 1 + min({d}_{x}, {d}_{y}) + (\widehat{d}/2) [(\widehat{d}/2) + (\widehat{d}\%2) - 1]$$

where $i$ is the node to be labeled, $x$ and $y$ are the target nodes, ${d}_{x}$ and ${d}_{y}$ represent the distance between $i$ and $x$ and between $i$ and $y$, respectively, $\widehat{d} = {d}_{x} + {d}_{y}$, $(\widehat{d}/2)$ is the quotient divided by 2, and $(\widehat{d}\%2)$ is the modulo operation divided by 2. If $i$ cannot reach $x$ or $y$, then $i$ is assigned a null label, 0. This method labels x and y as 1. The node labels are converted into one-hot encoding vectors.

### Feature extraction by GCN

We adopted a GCN^21^ to capture the structural features of the labeled nodes in the subgraph, which resulted in node representations. The GCN extracts not only local features but also global features, allowing us to deal with diverse features in the topology. The GCN updates hidden states as follows:

$${X}^{(l+1)} = GCN(A, {X}^{(l)}) = \sigma ({\widetilde{D}}^{-\frac{1}{2}}\widetilde{A}{\widetilde{D}}^{-\frac{1}{2}}{X}^{(l)}{W}^{(l)})$$

where ${X}^{(l+1)}$ is the result of the $l$ th GCN layer in the matrix form of the number of nodes by the number of features, $A$ is the adjacency matrix of the input subgraph, ${X}^{(l)}$ is the results of the previous GCN layer, $\sigma$ is the activation function, $\widetilde{A}$ is the adjacency matrix adding self-loops calculated in the form of $\widetilde{A} = A + I$, $I$ is the identity matrix, $\widetilde{D}$ is the diagonal matrix whose elements represent node degrees, ${W}^{(l)}$ is the trainable matrix at the $l$ th layer.

### Random walk profile generation by attention

A random walk algorithm computes transition probabilities of nodes based on edge weights in a graph. Unlike conventional methods, we used attention mechanisms to quantify which specific nodes will be emphasized based on the connectivity between nodes. The resulting attention scores improve the quality of the transition probabilities. The attention score ${\omega }_{i, j}$ between nodes $i$ and $j$ is calculated as follows:

$${\omega }_{i, j} = {{Q}_{\theta }({z}_{i})}^{T}{K}_{\theta }({z}_{j})/\sqrt{{N}_{att}}$$

where ${Q}_{\theta }$ is the query function, ${K}_{\theta }$ is the key function, ${z}_{i}$ and ${z}_{j}$ are the features of $i$ and $j$ computed from the GCN layer, respectively, and ${N}_{att}$ is the number of output dimensions of attention. This equation is also known as the value function in attention mechanisms. The attention scores between the nodes from this equation are encoded into the transition matrix $P$. The ($i$, $j$)-element of $P$, ${p}_{i, j}$, which indicates the transition probability from *i* to *j*, is computed as follows:

$${p}_{i, j} = { [softmax({({\omega }_{i, k})}_{k\in \mathcal{N}(i)})]}_{j} := exp({\omega }_{i, j}) / {\sum }_{k\in \mathcal{N}(i)}exp({\omega }_{i, k})$$

where $\mathcal{N}(i)$ is the set of neighbors of $i$. ${p}_{i, j}$ can be defined when $i$ and $j$ are linked. If $i$ and $j$ are not linked, then ${p}_{i, j}$ is zero. This framework adopts multi-head attention, as has been used in most previous studies for attention mechanisms. We applied 2-head attention in our experiment.

The $\tau$-th power of $P$, ${P}^{\tau }$, refers to the probability that a random walker will arrive at a walk of length-$\tau$ from node to node. We gather node-level, edge-level, and graph-level features for the random walk profile at length-$\tau$ as follows:

$${node}^{\tau } = {\left[{P}^{\tau }\right]}_{x, x} + {\left[{P}^{\tau }\right]}_{y, y}, {edge}^{\tau } = {\left[{P}^{\tau }\right]}_{x, y} + {\left[{P}^{\tau }\right]}_{y, x}, {graph}^{\tau } = tr\left[{P}^{\tau }\right]$$

where $x$ and $y$ are the nodes linked by the target edge for prediction. Node-level features represent loop structures around $x$ and $y$. Because we are dealing with undirected graphs, the summation of the node-level features guarantees that they are independent of the ordering of $x$ and $y$. Edge-level features describe the probability of a random walker reaching a target edge. Graph-level features are related to the self-loop probability of all nodes in the graph.

Node pairs without an edge are always considered negative for training and prediction. More precisely, during the training phase, a target link must always be present for a positive sample to be learned, and a target link must always be absent for a negative sample to be learned. Predictions, however, have to be made without a target link at all times during the prediction phase. This unfavorable situation typically causes overfitting. Thus, we train two distinct types of graphs: one includes the target link for prediction, while the other excludes the target link. These two different graphs are represented as ${{\text{G}}}^{+}$ and ${{\text{G}}}^{-}$, respectively. This data augmentation technique was applied to prevent overfitting. The attention mechanisms that are conducted in the two graphs, ${G}^{+}$ and ${G}^{-}$, create transition matrices ${P}^{+}$ and ${P}^{-}$ and random walk profiles including ${node}^{\tau , +}$, ${node}^{\tau , -}$, ${edge}^{\tau , +}$, ${edge}^{\tau , -}$, ${graph}^{\tau , +}$, and ${graph}^{\tau , -}$. However, a trace operation for graph-level features disturbs the structural information around the target link, making it unsuitable for link prediction. To solve this problem, WalkPooling^22^ used the “background subtraction” technique defined as $\Delta {graph}^{\tau } = {graph}^{\tau , +} - {graph}^{\tau , -}$. Finally, the features are concatenated as:

$$DRAW(G, Z) = \left[{\omega }_{i, j}, {\left({node}^{\tau , +}, {node}^{\tau , -},{edge}^{\tau , +}, {edge}^{\tau , -} ,\Delta {graph}^{\tau }\right)}_{\tau =2}^{{\tau }_{c}}\right]$$

where $G$ is the subgraph enclosing the target link, $Z$ is the features of G computed by GCN, and ${\tau }_{c}$ is the maximum walk length. In our experiment, we applied ${\tau }_{c}=7$ as a default. This equation is employed for each attention head. In the case of adopting multi-head attention as mentioned above, the resultant feature space forms the size of $num\_heads \times \left((5 \times {\tau }_{c}) + 1\right)$.

### Subgraph classification by MLP

The random walk profiles computed in the above steps are fed into a multi-layer perceptron (MLP) to predict whether a target link exists. An MLP consists of an input layer, four hidden layers, and an output layer. The ReLU function was adopted for activation through the hidden layers, and the sigmoid function was adopted for activation in the output layer.

## Experimental data

### Drug similarity networks

DrugBank^27^ was used to collect the drug datasets. This database includes a wide range of drug-related features, such as drug indications, drug targets, chemical structures, and drug-drug interactions. In this study, similarities between drugs were calculated using two salient characteristics: chemical structures and ATC codes. First, drug structural similarity was determined using simplified molecular-input line-entry specification (SMILES)^28^, which is a line notation system used to represent chemical compound structures. The Chemistry Development Kit (CDK)^29^ was employed to convert a pair of structures in SMILES format into a Tanimoto similarity score. Second, the similarities between drugs were measured based on their ATC codes^30^, a system to classify drugs in a hierarchy of pharmacological, therapeutic, and chemical categories. The similarity between ATC codes was calculated as follows:

$${{\text{sim}}}_{{\text{ATC}}}\left({{\text{ATC}}}_{{\text{i}}}, {{\text{ATC}}}_{{\text{j}}}\right) = \frac{{\text{C}}\left({{\text{ATC}}}_{{\text{i}}}\right) \cap {\text{C}}\left({{\text{ATC}}}_{{\text{j}}}\right)}{{\text{C}}\left({{\text{ATC}}}_{{\text{i}}}\right) \cup {\text{C}}\left({{\text{ATC}}}_{{\text{j}}}\right)}$$

where $ATC$ indicates each ATC code, and $C\left(ATC\right)$ is the set of codes from all ATC levels. It is noted that a drug may have multiple ATC codes, thus we used the average similarity of all ATC code pairs to calculate the similarity between drugs as follows:

$${{\text{sim}}}_{{\text{dr}}}\left({{\text{dr}}}_{{\text{x}}}, {{\text{dr}}}_{{\text{y}}}\right) = \frac{\sum_{{\text{i}}, {\text{j}}}{{\text{sim}}}_{{\text{ATC}}}\left({{\text{X}}}_{{\text{i}}}, {{\text{Y}}}_{{\text{j}}}\right)}{\left|{\text{X}}\right|*\left|{\text{Y}}\right|}$$

where $dr$ represents each drug, $X$ and $Y$ indicate the sets of ATC codes of each drug, and $\left|X\right|$ and $\left|Y\right|$ is the size of $X$ and $Y$, respectively. Each of the two drug similarity networks was merged with the disease similarity network and drug-disease associations to create two different heterogeneous networks, named network-CS and network-ATC.

### Disease similarity networks

Online Mendelian Inheritance in Man (OMIM)^31^ is an extensive collection of human genes and genetic diseases. It is continuously updated with a focus on disease-associated genes. To measure similarities between diseases, many previous studies have used MimMiner^32^, which provides a convention for representing phenotype networks. However, to analyze and quantify the relationships between diseases more accurately, we employed an ontology, which is a conceptual representation of entities with a standardized structure that links them based on the relationships between their meanings. We used Human Phenotype Ontology (HPO)^33^, a comprehensive phenotype ontology consisting of phenotypic abnormality terms linked by parent–child relationships. HPO also provides human disease annotations originating from the OMIM^31^, OrphaNet^34^, and DECIPHER^35^ databases. For our experiment, diseases from OMIM were extracted from HPO annotations. We adopted an approach to measure semantic similarity as suggested previously^36^. The following describes the calculation of the semantic similarity between two diseases:

$$sim({di}_{1}, {di}_{2}) = \frac{{\sum }_{{C}_{i}\in T({di}_{1})}{max}_{{C}_{j}\in T({di}_{2})} {sim}_{T}({C}_{i}, {C}_{j}) + {\sum }_{{C}_{j}\in T({di}_{2})}{max}_{{C}_{i}\in T({di}_{1})} {sim}_{T}({C}_{i}, {C}_{j})}{\left|T({d}_{1})\right| + \left|T({d}_{2})\right|}$$

where ${d}_{1}$ and ${d}_{2}$ are diseases annotated to HPO terms and $T(d)$ is the set of HPO terms annotating $d$, Note that a single disease can be annotated to multiple HPO terms. Finally, ${sim}_{T}$ denotes the semantic similarity between the two HPO terms ${C}_{i}$ and ${C}_{j}$, which is calculated as follows:

$${sim}_{T}({C}_{1}, {C}_{2}) = \frac{{\sum }_{{C}_{i}\in {A}_{t}({C}_{1})\cap {A}_{t}({C}_{2})}log P({C}_{i})}{{\sum }_{{C}_{j}\in {A}_{t}({C}_{1})\cup {A}_{t}({C}_{2})}log P({C}_{j})}$$

where ${A}_{t}(C)$ is a set of ancestor terms of $C$ and $P(C)$ is the ratio of annotations as the number of annotations to $C$ over the number of annotations to all terms in HPO.

### Drug-disease associations

The Cdataset^37^ is one of the most widely used benchmark datasets for drug-disease associations in recent drug repositioning research. This benchmark, which contains 663 drugs, 409 diseases, and 2352 drug-disease associations, is an upgraded version of the initial ground-truth, Fdataset^38^, and was created by appending clinically validated data from the DNdataset^10^. From the Cdataset, we selected only drugs and diseases available in the constructed drug and disease similarity networks. Finally, 659 drugs, 285 diseases, and 1728 drug-disease associations were selected for network-CS, and 636 drugs, 285 diseases, and 1681 drug-disease associations were selected for network-ATC, as shown in Table 1.

**Table 1:** Statistics of the two datasets that we used for our experiment on drug-disease association prediction.

	Number of drugs	Number of diseases	Number of associations	Sparsity
Network-CS	659	285	1728	$9.20\times {10}^{-3}$
Network-ATC	636	285	1681	$9.27\times {10}^{-3}$

## Results

### Experimental setting

The predictive results of drug-disease associations were assessed individually on the drug side and disease side. Prediction on the drug side identifies new diseases that each medication could treat, whereas prediction on the disease side identifies medications with the potential to treat each disease. We applied 10-fold cross-validation for this assessment. The folds were evenly divided based on the number of drugs, diseases, and their associations to ensure impartial analysis.

While the sigmoid function in the output layer is typically applied for binary classification, for ranking potential target links, we opt not to use the sigmoid activation, thereby leveraging the model’s raw scores. To maintain the integrity of the training process and ensure a balanced representation, negative samples were selected to mirror the quantity of positive training data, explicitly excluding any instances from the test dataset. The optimization of this model is guided by minimizing the binary cross-entropy loss function.

The AUC was used to compare predictive performance. An ROC curve was created by plotting the true positive rate (TPR) against the false positive rate (FPR) as the threshold settings were changed. The AUC is typically regarded as the most effective metric for quantifying predictive power. We also used AUPR*, a transformed version of the area under the precision-recall curve (AUPR) described previously^11^. The precision-recall curve plots precision against recall as the threshold settings change. However, drug-disease associations were remarkably sparse in our experimental dataset. This generally causes very low precision because of the extremely large number of false positives (FP) compared to true positives (TP), where precision is $TP / (TP + FP)$. To resolve the biased results from AUPR, we adopted AUPR* using precision* instead of precision, where precision* was defined as $TPR / (TPR + FPR)$.

### Predictive accuracy comparison

Recent network-based approaches for drug-disease association prediction can be divided into three categories: graph-mining algorithms, matrix factorization, and deep learning models. In this section, we compare the predictive performance of DRAW with that of five state-of-the-art methods: three methods using deep-learning models (deepDR, ANMF, and LAGCN), and the most recent methods in the other two categories (BGMSDDA and MSBMF), as listed below. The best hyperparameter values recommended in the previous studies were used to implement each method. To compare the predictive performance, we applied 10-fold cross-validation for all the methods.

- BGMSDDA^39^ applies a graph diffusion technique to a bipartite graph that integrates multiple similarities using Gaussian interaction profiles.
- MSBMF^40^ is based on bi-linear matrix factorization using multiple similarities as latent features.
- deepDR^41^ constructs multiple positive point-wise mutual information (PPMI) matrices from multiple sources, and applies a multi-modal deep autoencoder (MDA) to combine these matrices.
- ANMF^42^ uses an autoencoder with the similarities including Gaussian noise to extract the features of drugs and diseases.
- LAGCN^43^ adopts the attention mechanism for layers in a GCN to predict drug-disease associations.

**Figure 2:**
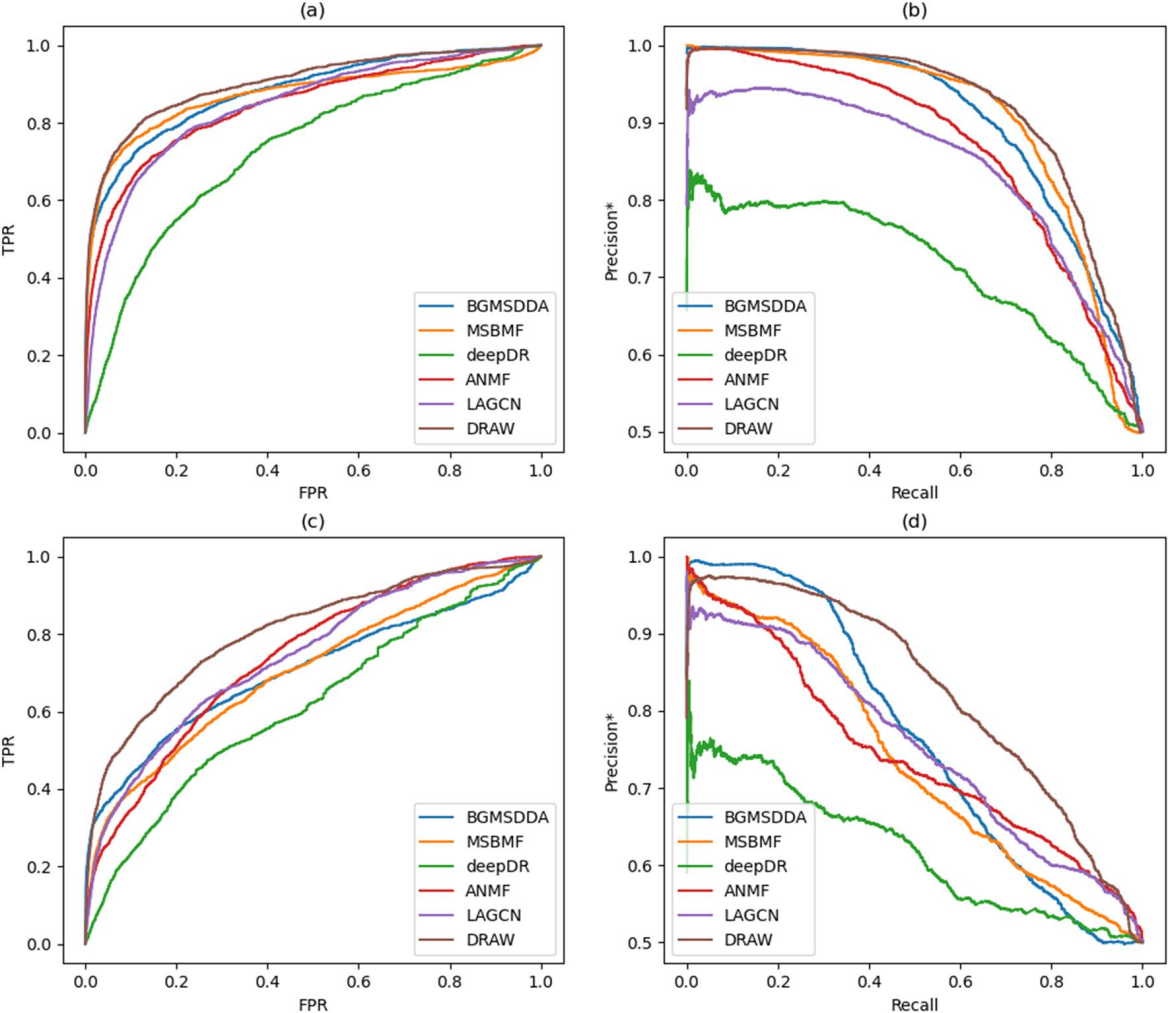
Accuracy comparison of the proposed method, DRAW, and the five state-of-the-art methods for drug-disease association prediction when network-ATC is used: (a) ROC curves on the drug-side, (b) Precision*-recall curves on the drug-side, (c) ROC curves on the disease-side, and (d) Precision*-recall curves on the disease-side.

Table 2 shows the predictive accuracy of the selected methods in terms of AUC and AUPR* when network-ATC was used. The proposed method, DRAW, had the highest AUC and AUPR* in both drug and disease side predictions. On the drug side, the AUC of DRAW was 0.903, which was 2.5% higher than that of the second-ranked BGMSDDA. DRAW also had the highest AUPR* score (0.915). On the disease side, the gap between DRAW and the second class widened. DRAW achieved an AUC of 0.807, which was 8.8% higher than that of the second-ranked LAGCN, and an AUPR* of 0.807, which was 7.7% higher than that of the second-ranked BGMSDDA. Figure 2 shows the ROC and precision*-recall curves for predicting drug-disease associations with network-ATC. These curves verify that DRAW was superior to the other methods, especially for disease side prediction as shown in Figs. 2c and d.

**Table 2:** Accuracy comparison for drug-disease association prediction by 10-fold cross-validation when network-ATC is used.

Method	prediction on the drug-side		prediction on the disease-side	
	AUC	AUPR*	AUC	AUPR*
BGMSDDA	0.881	0.701	0.705	0.765
MSBMF	0.872	0.902	0.702	0.735
deepDR	0.730	0.714	0.614	0.62
ANMF	0.845	0.868	0.739	0.743
LAGCN	0.842	0.843	0.742	0.753
DRAW (the proposed)	**0.903**	**0.915**	**0.807**	**0.824**

The highest score in each evaluation category is in bold.

**Figure 3:**
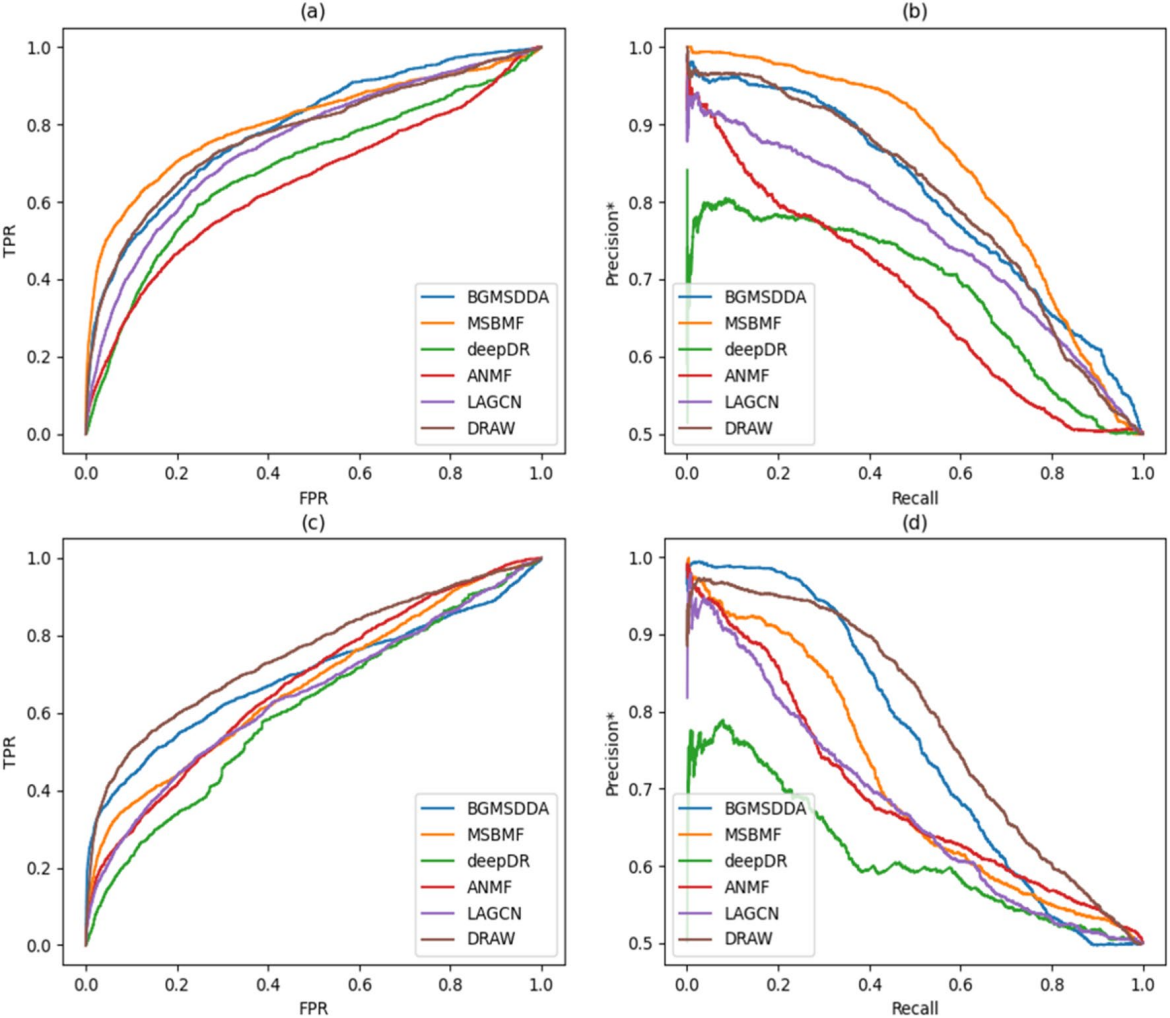
Accuracy comparison of the proposed method, DRAW, and the five state-of-the-art methods for drug-disease association prediction when network-CS is used: (**a**) ROC curves on the drug-side, (**b**) Precision*-recall curves on the drug-side, (**c**) ROC curves on the disease-side, and (**d**) Precision*-recall curves on the disease-side.

Table 3 shows the predictive accuracy of the selected methods in terms of AUC and AUPR* when network-CS was used. For disease side prediction, DRAW performed better than the other methods. DRAW achieved an AUC of 0.752 and an AUPR* of 0.784, which were 8.3% and 3.2% higher, respectively, than those of the second-ranked BGMSDDA. However, for drug side prediction, DRAW had slightly lower accuracy than BGMSDDA, which is a graph-mining algorithm, and MSBMF, which is a matrix factorization algorithm, in terms of both AUC and AUPR*. Figure 3 shows the ROC and precision*-recall curves for predicting drug-disease associations with network-CS. Figure 3c and d show that DRAW was more accurate than the others for disease side prediction, whereas BGMSDDA was better than DRAW for drug side prediction in Fig. 3a and b.

**Table 3:** Accuracy comparison for drug-disease association prediction by 10-fold cross-validation when network-CS is used.

Method	prediction on the drug-side		prediction on the disease-side	
	AUC	AUPR*	AUC	AUPR*
BGMSDDA	0.790	0.804	0.694	0.760
MSBMF	**0.805**	**0.842**	0.669	0.708
deepDR	0.685	0.686	0.606	0.613
ANMF	0.646	0.678	0.673	0.692
LAGCN	0.751	0.756	0.643	0.677
DRAW (the proposed)	0.774	0.800	**0.752**	**0.784**

The highest score in each evaluation category is in bold.

Overall, DRAW outperformed the competitive methods. However, the previously proposed deep learning methods generally showed slightly lower accuracy than the graph mining and matrix factorization algorithms, particularly for drug side prediction. Typically, approaches based on deep learning models are highly sensitive to the quality and quantity of input data. For this reason, the deep learning methods, including DRAW, showed relatively low predictive accuracy on the drug side with network-CS, although DRAW always achieved first place among the deep learning methods selected for this experiment.

### Effects of network density

**Figure 4:**
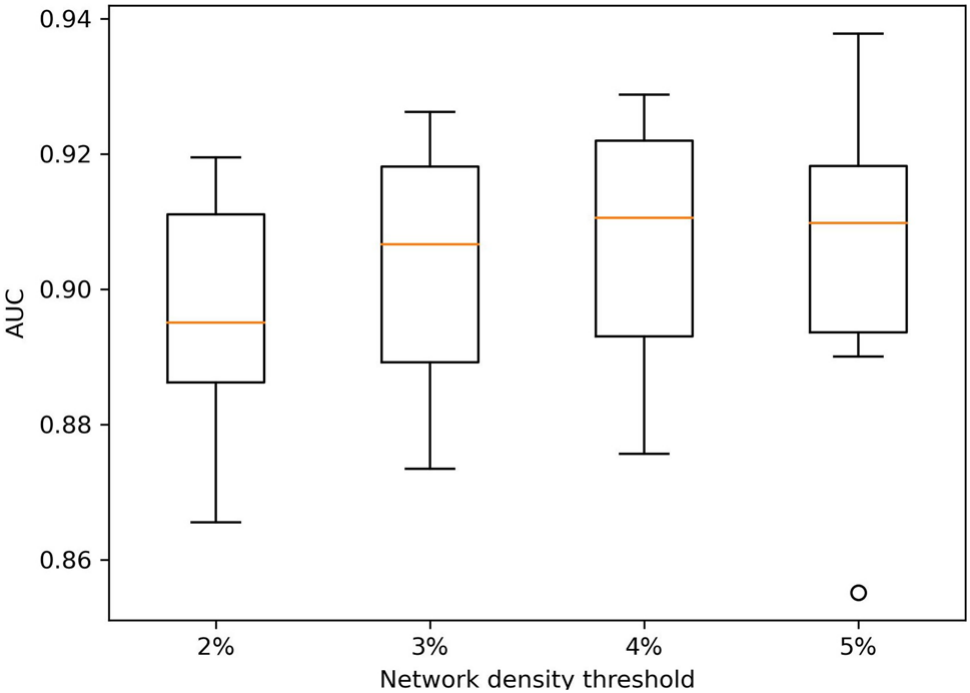
The distributions of AUC values from 10 folds when the network density thresholds are 2%, 3%, 4%, and 5%. Drug-disease associations were predicted on the drug side with network-ATC by DRAW. The highest median AUC was achieved when the density threshold was 4%.

In network-based approaches for drug-disease association prediction, the density of input networks significantly affects predictive accuracy. We assessed the effect of the density of the drug and disease networks in our 10-fold cross-validation experiments. Figure 4 shows the distributions of AUC values from 10 folds when DRAW predicts drug-disease associations on the drug side with network-ATC. The four boxplots in this figure show the results when the network density thresholds were 2%, 3%, 4%, and 5%. The highest median AUC was achieved when the density threshold was 4%, indicating that the densities of both the drug and disease networks were 4%. When the density threshold is 5%, the median AUC decreased, and AUC values were more widely dispersed, including an AUC lower than 0.86 as an outlier. Selecting a higher density threshold implies that the input network contains more edges with lower similarity scores. It can be verified that selecting a density threshold higher than 4% negatively affects predictive performance. Therefore, we used a density threshold of 4% in our experiments.

## Case studies

In this section, we present exploratory case studies of drug repositioning for specific drugs and diseases. We created a training set including all known drug-disease associations and a test set comprising the other drug-disease pairs in network-ATC. DRAW learned the training set to extract features and computed the prediction scores for drug-disease pairs in the test set. For each drug, all diseases were listed in descending order of their prediction scores. For each disease, all drugs were administered in the same manner. Finally, we validated the results using publicly available databases, such as The Comparative Toxicogenomics Database (CTD)^44^, DrugBank^27^, and KEGG^45^. Tables 4, 5, 6 and 7 show the high-ranked prediction results for selected drugs: doxorubicin, gabapentin, levodopa, and flecainide. From these results, we identified 10 evidences out of 10 for doxorubicin, 8 out of 9 for gabapentin, 6 out of 9 for levodopa, and 6 out of 8 for flecainide. Tables 8, 9, 10 and 11 show the high-ranked prediction results for selected diseases: type 2 diabetes mellitus, ischemic stroke, Alzheimer’s disease, and Parkinson’s disease. From these results, we identified 9 evidences out of 10 for type 2 diabetes mellitus, 6 out of 9 for ischemic stroke, 8 out of 10 for Alzheimer’s disease, and 8 out of 10 for Parkinson’s disease.

**Table 4:** The top 10 candidate diseases to be treated by doxorubicin.

Rank	Candidate disease (OMIM ID)	Evidence
1	Small cell cancer of the lung (182,280)	CTD
2	Dohle bodies and leukemia (223,350)	CTD
3	Testicular germ cell tumor; TGCT (273,300)	CTD
4	Myeloproliferative disorder, chronic, with eosinophilia (131,440)	CTD
5	Kaposi sarcoma, susceptibility to (148,000)	KEGG
6	Hypereosinophilic syndrome, idiopathic; HES (607,685)	CTD
7	Glioma Susceptibility 1; GLM1 (137,800)	CTD
8	Leukemia, acute myelocytic, with polyposis coli and colon cancer (264,670)	CTD
9	Prostate cancer (176,807)	CTD
10	Pheochromocytoma (171,300)	CTD

**Table 5:** The top 9 candidate diseases to be treated by gabapentin.

Rank	Candidate disease (OMIM ID)	Evidence
1	Hyperphosphatemia, polyuria, and seizures (239,350)	CTD/DrugBank
2	Myoclonic epilepsy, familial infantile; FIME (605,021)	CTD/DrugBank
3	Epilepsy, myoclonic juvenile; EJM (254,770)	CTD/DrugBank
4	Seizures, benign familial neonatal, 2; BFNS2 (121,201)	CTD/DrugBank
5	Macrocephaly and epileptic encephalopathy (606,369)	
6	Seizures, benign familial neonatal, 1; BFNS1 (121,200)	CTD
7	Acromegaloid changes, cutis verticis gyrate, and corneal leukoma (102,100)	CTD
8	Schizophrenia; SCZD (181,500)	CTD
9	Developmental and epileptic encephalopathy 1; DEE1 (308,350)	CTD

**Table 6:** The top 9 candidate diseases to be treated by levodopa.

Rank	Candidate disease (OMIM ID)	Evidence
1	Attention deficit-hyperactivity disorder; ADHD (143,465)	CTD
2	Insensitivity to pain with hyperplastic myelinopathy (147,530)	
3	Dementia; Parkinsonism with non-alzheimer amyloid plaques (125,320)	CTD/DrugBank
4	Hyperthermia, cutaneous, with headaches and nausea (145,590)	
5	Alcohol dependence (103,780)	CTD
6	Tremor, nystagmus, and duodenal ulcer (190,310)	
7	Alzheimer disease, familial, 1; AD1 (104,300)	CTD
8	Narcolepsy 1; NRCLP1 (161,400)	CTD
9	Alzheimer disease 4 (606,889)	CTD

**Table 7:** The top 8 candidate diseases to be treated by flecainide.

Rank	Candidate disease (OMIM ID)	Evidence
1	Ventricular arrhythmias due to cardiac ryanodine receptor calcium release deficiency syndrome; VACRDS (115,500)	CTD/DrugBank
2	Cataract, aberrant oral frenula, and growth retardation (115,645)	
3	Renal failure, progressive, with hypertension; RFH1 (161,900)	CTD
4	Portal vein, cavernous transformation of (601,004)	
5	Insensitivity to pain with hyperplastic myelinopathy (147,530)	CTD
6	Cerebral arteriopathy, autosomal dominant, with subcortical infarcts and leukoencephalopathy, type 1; CADASIL1 (125,310)	
7	Renal cell carcinoma, nonpapillary; RCC (144,700)	CTD
8	Heart block, congenital (234,700)	CTD

**Table 8:** The top 10 candidate medications to treat type 2 diabetes mellitus.

Rank	Candidate drug	Evidence
1	Canagliflozin	CTD
2	Lansoprazole	CTD
3	Simvastatin	CTD
4	Orlistat	CTD
5	Diethylpropion	
6	Pantoprazole	CTD
7	Omeprazole	CTD
8	Rosuvastatin	CTD
9	Sibutramine	CTD
10	Metamfetamine	CTD

**Table 9:** The top 9 candidate medications to treat Ischemic stroke.

Rank	Candidate drug	Evidence
1	Ticlopidine	CTD
2	Tirofiban	
3	Fondaparinux	
4	Epoprostenol	CTD
5	Lisinopril	CTD
6	Isradipine	CTD
7	Hydrocodone	
8	Nisoldipine	CTD
9	Norepinephrine	CTD

**Table 10:** The top 10 candidate medications to treat Alzheimer’s disease.

Rank	Candidate drug	Evidence
1	Pramipexole	CTD
2	Procyclidine	CTD
3	Ropinirole	CTD
4	Trihexyphenidyl	CTD
5	Scopolamine	CTD
6	Apomorphine	
7	Benzatropine	CTD
8	Levodopa	CTD
9	Orphenadrine	CTD
10	Lisuride

**Table 11:** The top 10 candidate medications to treat Parkinson’s disease.

Rank	Candidate drug	Evidence
1	Amantadine	CTD
2	Bromocriptine	CTD
3	Meprobamate	
4	Ondansetron	CTD
5	Galantamine	CTD
6	Biperiden	CTD
7	Memantine	CTD
8	Donepezil	CTD
9	Tacrine	CTD
10	Flavoxate	

The drugs listed in Table 8 serve therapeutic purposes across various medical conditions, including gastrointestinal disorders (ranked 2, 6 and 7), hypercholesterolemia (ranked 3 and 8), and obesity (ranked 4, 5, and 9). Canagliflozin, initially indicated for type-1 diabetes and occupying the first rank, has been used for type-2 diabetes management. Recent advancements have extended the utility of several medications originally intended for diabetes treatment, such as liraglutide and semaglutide, to address obesity. These results provide insights into the appropriateness of the pharmaceuticals listed by our model, in accordance with evolving therapeutic trends.

The drugs to treat ischemic stroke in Table 9 belong to several groups, including blood pressure management, antithrombotic agents, antiplatelet medications, and vasodilators. These medications play a crucial role in reducing the risks associated with ischemic stroke, which occurs when blood flow to the brain is blocked or reduced. The strong correlation between these drugs and ischemic stroke underscores their significance in managing this condition. Furthermore, most of the medications listed in Tables 10 and 11 are known as treating Parkinson’s disease and Alzheimer’s disease, respectively. These results demonstrate the strong correlation between Parkinson’s and Alzheimer’s diseases.

## Discussion and conclusion

Computational drug repositioning is a promising research area because it remarkably reduces the time, costs, and risk associated with traditional drug discovery. Particularly, network-based computational approaches have widely been applied because they can effectively predict and validate drug-disease associations in a system level. In this article, we presented a novel method to predict drug-disease associations using a drug-disease heterogeneous network. Unlike other methods, our approach adopted a random walk algorithm and recalculated transition probabilities based on attention mechanisms. The experimental results revealed that the proposed method outperformed state-of-the-art methods. DRAW also had substantially higher predictive accuracy than the deep learning algorithms proposed previously. The proposed model has several significant advantages. First, it demonstrates efficiency in memory usage by conducting a random walk on a subgraph enclosing each pair for association prediction, rather than on an entire heterogeneous network. Consequently, it is applicable to large networks on an omics scale. Second, our model eliminates the need for re-training even when new drugs or diseases are added because it takes a subgraph as input, regardless of the number of nodes and edges.

Our experimental results showed that all methods performed better for drug side prediction rather than disease side prediction regardless of the input network. This suggests that computational drug repositioning may be better suited for identifying additional diseases that can be treated by new drugs. This result might be obtained because of inaccuracy of the measured similarities between drugs, or the unbalanced numbers of drugs and diseases in the input network. Nevertheless, DRAW had the highest predictive accuracy on the disease side.

All methods in our experiment also performed better with the network constructed by the similarities based on ATC codes rather than that by structural similarities. Utilizing ATC codes for classifying drug-disease associations leverages the therapeutic and pharmacological properties of their active ingredients, offering a more relevant measure of efficacy than chemical structure analysis. Furthermore, because the hyperparameter of network density was optimized on 4\% in our experiment, it was validated that incorporating higher densities, i.e., including connections with lower similarity scores, detracts from the effectiveness of network-based methodologies. In other words, a limited number of drug or disease pairs with high similarities provide sufficient information for drug-disease association prediction.

Several future directions for this research are suggested to enhance the effectiveness of computational drug repositioning. First, multiple biological, therapeutic features regarding diseases and medications can be integrated to improve the predictive accuracy of drug-disease associations. In particular, the integration with additional data of drug-target interactions might have a great influence on association prediction. Because of recent active research of drug-target interaction prediction, the number of open-source databases containing putative drug targets has been rapidly increased, such as DrugBank^27^, BindingDB^46^, SuperTarget^47^, and STITCH^48^. Next, the proposed model might be improved further by discriminating between node types in a drug-disease heterogeneous network. For example, node2vec^49^, one of the most widely used node embedding methods, did not differentiate between node types. However, HIN2vec^50^, an extension of node2vec, facilitated performance improvement by including the features of the graph heterogeneity.

## Data Availability

The source code is available at https://ads.yonsei.ac.kr/DRAW.

## References

[1] Li J (2016). A survey of current trends in computational drug repositioning. Brief. Bioinform..

[2] Paul SM (2010). How to improve R&D productivity: The pharmaceutical industry’s grand challenge. Nat. Rev. Drug Discov..

[3] Pushpakom S (2019). Drug repurposing: Progress, challenges and recommendations. Nat. Rev. Drug Discov..

[4] Chan HS, Shan H, Dahoun T, Vogel H, Yuan S (2019). Advancing drug discovery via artificial intelligence. Trends Pharmacol. Sci..

[5] Dickson M, Gagnon JP (2004). Key factors in the rising cost of new drug discovery and development. Nat. Rev. Drug Discov..

[6] Hurle MR (2013). Computational drug repositioning: From data to therapeutics. Clin. Pharmacol. Ther..

[7] Ashburn T, Thor K (2004). Drug repositioning: Identifying and developing new uses for existing drugs. Nat. Rev. Drug Discov..

[8] Luo H (2021). Biomedical data and computational models for drug repositioning: A comprehensive review. Brief. Bioinform..

[9] Zhao Q, Yu H, Ji M, Zhao Y, Chen X (2019). Computational model development of drug-target interaction prediction: A review. Curr. Pro. Pept. Sci..

[10] Martinez V, Navarro C, Cano C, Fajardo W, Blanco A (2015). DrugNet: Network-based drug–disease prioritization by integrating heterogeneous data. Artif. Intel. Med..

[11] Kim Y, Jung YS, Park JH, Kim SJ, Cho YR (2022). Drug-disease association prediction using heterogeneous networks for computational drug repositioning. Biomolecules.

[12] He J, Yang X, Gong Z (2020). Hybrid attentional memory network for computational drug repositioning. BMC Bioinformatics.

[13] Koren Y, Bell R, Volinsky C (2009). Matrix factorization techniques for recommender systems. Computer.

[14] Liu H, Kou H, Yan C, Qi L (2019). Link prediction in paper citation network to construct paper correlation graph. EURASIP J. Wirel. Commun. Netw..

[15] Kovács IA (2019). Network-based prediction of protein interactions. Nat. Commun..

[16] Adamic LA, Adar E (2003). Friends and neighbors on the web. Soc. Netw..

[17] Katz L (1953). A new status index derived from sociometric analysis. Psychometrika.

[18] Brin S, Page L (1998). The anatomy of a large-scale hypertextual web search engine. Comput. Netw. ISDN Syst..

[19] Scarselli F, Gori M, Tsoi AC, Hagenbuchner M, Monfardini G (2008). The graph neural network model. IEEE Trans. Neural Netw..

[20] Zhou J (2020). Graph neural networks: A review of methods and applications. AI Open.

[21] Kipf, T. N., & Welling, M. Semi-supervised classification with graph convolutional networks. *In Proc. 5th International Conference on Learning Representations (ICLR)* (2017).

[22] Pan, L., Shi, C., & Dokmanić, I. Neural link prediction with walk pooling. *In Proc. 10th International Conference on Learning Representations (ICLR)* (2022).

[23] LeCun Y (1989). Backpropagation applied to handwritten zip code recognition. Neural Comput..

[24] Bahdanau, D., Cho, K., & Bengio, Y. Neural machine translation by jointly learning to align and translate. *In Proc. 3rd International Conference on Learning Representations (ICLR)* (2015).

[25] Gardner MW, Dorling SR (1998). Artificial neural networks (the multilayer perceptron)—A review of applications in the atmospheric sciences. Atmos. Environ..

[26] Zhang, M., & Chen, Y. Link prediction based on graph neural networks. *In Proc. 32nd Conference on Neural Information Processing Systems (NIPS)* (2018).

[27] Wishart DS (2018). DrugBank 5.0: A major update to the DrugBank database for 2018. Nucleic Acids Res..

[28] Weininger D (1988). SMILES a chemical language and information system. 1. Introduction to methodology and encoding rules. J. Chem. Inf Comput Sci.

[29] Steinbeck C (2003). The chemistry development kit (CDK): An open-source Java library for chemo-and bioinformatics. J. Chem. Inf Comput Sci.

[30] Olson T, Singh R (2017). Predicting anatomic therapeutic chemical classification codes using tiered learning. BMC Bioinformatics.

[31] Amberger JS, Bocchini CA, Scott AF, Hamosh A (2019). OMIM.org: Leveraging knowledge across phenotype–gene relationships. Nucleic Acids Res..

[32] Van Driel MA, Bruggeman J, Vriend G, Brunner HG, Leunissen JA (2006). A text-mining analysis of the human phenome. Eur. J. Hum. Genet..

[33] Köhler S (2021). The human phenotype ontology in 2021. Nucleic Acids Res..

[34] Wakap SN (2020). Estimating cumulative point prevalence of rare diseases: Analysis of the orphanet database. Eur. J. Hum. Genet..

[35] Bragin E (2014). DECIPHER: Database for the interpretation of phenotype-linked plausibly pathogenic sequence and copy-number variation. Nucleic Acids Res..

[36] Pesquita C, Faria D, Falcao AO, Lord P, Couto FM (2009). Semantic similarity in biomedical ontologies. PLoS Comput. Biol..

[37] Luo H (2016). Drug repositioning based on comprehensive similarity measures and bi-random walk algorithm. Bioinformatics.

[38] Gottlieb A, Stein GY, Ruppin E, Sharan R (2011). PREDICT: A method for inferring novel drug indications with application to personalized medicine. Mol. Syst. Biol..

[39] Xie G (2021). BGMSDDA: A bipartite graph diffusion algorithm with multiple similarity integration for drug–disease association prediction. Mol. Omics.

[40] Yang M, Wu G, Zhao Q, Li Y, Wang J (2021). Computational drug repositioning based on multi-similarities bilinear matrix factorization. Brief. Bioinform..

[41] Zeng X (2019). deepDR: A network-based deep learning approach to in silico drug repositioning. Bioinformatics.

[42] Yang X, Zamit L, Liu Y, He J (2019). Additional neural matrix factorization model for computational drug repositioning. BMC Bioinformatics.

[43] Yu Z, Huang F, Zhao X, Xiao W, Zhang W (2021). Predicting drug–disease associations through layer attention graph convolutional network. Brief. Bioinform..

[44] Davis AP (2021). Comparative toxicogenomics database (CTD): Update 2021. Nucleic Acids Res..

[45] Kanehisa M, Furumichi M, Tanabe M, Sato Y, Morishima K (2017). KEGG: New perspectives on genomes, pathways, diseases and drugs. Nucleic Acids Res..

[46] Gilson MK (2016). BindingDB in 2015: A public database for medicinal chemistry, computational chemistry and systems pharmacology. Nucleic Acids Res..

[47] Hecker N (2012). SuperTarget goes quantitative: Update on drug–target interactions. Nucleic Acids Res..

[48] Kuhn M (2014). STITCH 4: Integration of protein–chemical interactions with user data. Nucleic Acids Res..

[49] Grover, A. & Leskovec, J. node2vec: scalable feature learning for networks. *In Proc. ACM SIGKDD Int. Conference on Knowledge Discovery and Data Mining (KDD)* 855–864 (2016). 10.1145/2939672.2939754 PMC5108654 27853626

[50] Fu, T. Y., Lee, W. C., & Lei, Z. Hin2vec: Explore meta-paths in heterogeneous information networks for representation learning. *In Proc. ACM Conference of Inf. Knowl. Manage. (CIKM)* 1797–1806 (2017).

